# Senescence-associated reprogramming induced by interleukin-1 impairs response to EGFR neutralization

**DOI:** 10.1186/s11658-022-00319-7

**Published:** 2022-03-02

**Authors:** Donatella Romaniello, Valerio Gelfo, Federica Pagano, Enea Ferlizza, Michela Sgarzi, Martina Mazzeschi, Alessandra Morselli, Carmen Miano, Gabriele D’Uva, Mattia Lauriola

**Affiliations:** 1 Department of Experimental, Diagnostic and Specialty Medicine (DIMES), University of Bologna, 40138 Bologna, Italy; 2 Centre for Applied Biomedical Research (CRBA), Bologna University Hospital Authority St. Orsola-Malpighi Polyclinic, 40138 Bologna, Italy; 3 National Laboratory of Molecular Biology and Stem Cell Engineering, National Institute of Biostructures and Biosystems (INBB), Bologna, Italy

**Keywords:** EGFR, Pseudo-senescence, IL-1, Cell plasticity, Colon cancer, Cetuximab, Resistance

## Abstract

**Background:**

EGFR targeting is currently the main treatment strategy for metastatic colorectal cancer (mCRC). Results of different clinical trials show that patients with wild-type KRAS and BRAF benefit from anti-EGFR monoclonal antibodies (moAbs) cetuximab (CTX) or panitumumab. Unfortunately, despite initial response, patients soon became refractory. Tumor heterogeneity and multiple escaping routes have been addressed as the main culprit, and, behind genomic alterations already described, changes in signaling pathways induced by drug pressure are emerging as mechanisms of acquired resistance. We previously reported an association between reduced sensitivity to CTX and increased expression of IL-1. However, how IL-1 mediates CTX resistance in mCRC is still unclear.

**Methods:**

Under CTX treatment, the upregulation of IL-1R1 expression and a senescence program in sensitive colorectal cancer (CRC) cell lines is examined over time using qPCR, immunoblotting, and immunofluorescence.

**Results:**

In sensitive CRC cells, IL-1 appeared responsible for a CTX-mediated G0 phase arrest. On the contrary, CTX-resistant CRC cells (CXR) maintained high mRNA levels of IL-1R1 and a post-senescence reprogramming, as indicated by increased SNAIL expression. Interestingly, treatment of CXR cells with a recombinant decoy, able to sequester the soluble form of IL-1, pushed CTX-resistant CRC cells back into a stage of senescence, thus blocking their proliferation. Our model suggests a trans-regulatory mechanism mediated by IL-1 on EGFR signaling. By establishing senescence and regulating EGFR activity and expression, IL-1 exposure ultimately bestows resistance.

**Conclusions:**

To sum up, our findings point to the combined blockage of IL-1R and EGFR as a promising therapeutical approach to restore sensitivity to EGFR-targeting monoclonal antibodies.

**Supplementary Information:**

The online version contains supplementary material available at 10.1186/s11658-022-00319-7.

## Background

According to GLOBOCAN data, colorectal cancer (CRC) is the second most deadly cancer worldwide and its incidence is constantly growing in developing nations [1]. Considering its high molecular heterogeneity, the only personalized approach to treat metastatic colorectal cancer (mCRC) is limited to the mutational analyses of *RAS* genes [2]. Results of different clinical trials show that patients with wild-type KRAS and BRAF benefit from anti-EGFR monoclonal antibodies (moAbs), namely cetuximab (CTX) or panitumumab [3–8]. However, those tumors (in most cases, liver metastasis), after a massive initial reduction, rapidly regrow and become refractory to therapies. Behind intrinsic genetic alterations, mechanisms of acquired resistance to anti-EGFR treatments are also triggered by nonmutational events of reversible signaling induced by drug pressure [9–11]. Thus, compensatory activation of biochemical feedback circuits, transcriptional modifications, or microenvironment changes may contribute to therapeutic failure. In our previous work, we reported an association between reduced sensitivity to anti-EGFR antibodies and increased expression of interleukin-1 (IL-1). Specifically, in a dataset of 150 xenopatients with CRC, patients not responsive to CTX showed a higher abundance of the pro-inflammatory cytokines IL-1α, IL-1β, and IL-8. The same trend was confirmed in a human CRC cell line insensitive to CTX (Caco-2 CXR) [12]. Furthermore, an in silico analysis of gene expression profile of 1700 CRC patients, revealed an impressive association of IL-1 receptor 1 (IL-1R1) expression with poor survival [13]. However, how IL-1 mediates CTX resistance in CRC is still unclear. It is known that a pro-inflammatory environment is responsible for inducing cellular stress, which often triggers a senescence response (referred to as senescence-associated secretory phenotype, SASP) [14]. Senescence is generally considered as a safe program of insult response, namely DNA damage, oncogene activation, telomere shortening, and so on, in which cells irreversibly stop dividing and enter a state of permanent growth arrest with tumoral suppressive function. However, new findings demonstrate that senescence in tumor cells is actually a dynamic process and may contribute to cancer recurrence after therapy, a process known as “pseudo-senescence” or “senescence-like arrest” [15–17]. Herein, by using two different CRC cell lines, we described that CTX treatment induces IL-1 pathway activation, which initially drives cancer cell into a pseudo-senescence state with cell cycle arrest. Thereafter, paradoxically, a chronic IL-1 exposure would reprogram CTX-resistant cells to gradually re-enter into the cell cycle, acquiring a poorly differentiated phenotype. A designed human recombinant decoy neutralizing IL1α/β (TRAP IL-1) was employed in our experimental settings and proved able to revert CTX resistance CRC cell lines to a senescence-mediated growth arrest.

## Materials and methods

### Cell lines and establishment of resistant cells

In vitro experiments have been conducted in two different colorectal cancer (CRC) cell lines, SW48 and Caco-2. The SW48 cells were cultured in Leibovitz’s (L-15, PanBiotech) medium containing 10% fetal bovine serum (FBS) (Sigma), 1% penicillin/streptomycin (Corning), and 1% l-glutamine (Corning). Cells were grown in T-25 flasks and incubated at 37 °C without CO_2_. Caco-2 cells were cultured in Dulbecco’s Minimal Essential Medium (DMEM, Corning) containing 10% ofetal bovine serum (FBS) (Sigma) and 1% penicillin/streptomycin (Corning). The cells were grown in 10 cm plastic Petri dishes and incubated at 37 °C in a humidified atmosphere of 5% CO_2_/air. Caco-2 CXR and SW48 CXR cells were cultured adding CTX (Erbitux, Merck KgaA, Germany) at increasing concentrations for few months, till the resistant cells were established and then maintained in culture at 10 μg/ml and 50 µg/ml, respectively.

### Proliferation assay

Cells were seeded in 96-well plates in 10% of FBS medium. The following day, quantification of initial time (time 0) was performed adding Alamar Blue reagent (resazurin) and measuring the fluorescence after 4 h of incubation. Fluorescence was quantified using VICTOR2TM 1420 multilabel counter (Perkin Elmer, Massachusetts, USA), at a wavelength of 595 nm. Then, cells were treated according to the experiment and, after 5 days, proliferation was measured as described above. Data were analyzed, and the median for each treatment (in triplicates) was calculated and reported as percentage relative to the control.

### Colony forming assay

Cells were seeded in 12-well plates in 10% of FBS medium. Treatments were added the following day. After at least 10 days, the medium was removed, and the cells were washed with PBS and fixed with a solution of PFA 4% for 20 min at room temperature. After washing with PBS, cells were stained with a solution of crystal violet 0.5% for 30 min and washed with water to remove excess dye. Pictures of each well were taken, and the covered area was measured and quantified using ImageJ software.

### β-Galactosidase staining

Cells were seeded in 12-well plates in 10% of FBS medium and treated the following day. After 4 days, cells were washed twice with PBS and fixed using 1× fixative solution (#11674, Cell Signalling Technology) for a maximum of 15 min. Consequently, β-galactosidase staining was performed by incubating cells with senescence β-galactosidase KIT solution (#9860; Cell Signalling Technology) for 5 h at 37 °C without CO_2_. Photos were taken at 20× magnification (Leica), and the percentage of senescence-associated (SA) β-galactosidase-positive cells (stained in blue) was calculated from the total cell number.

### Immunofluorescence

Cells were seeded on glass coverslips previously inserted into a 24-well plate with 10% FBS medium. After 4 days of treatment, cells were washed in PBS and fixed in cold 4% paraformaldehyde for 20 min. Subsequently cells were blocked with 1% bovine serum albumin (BSA:PBS 1:1) for 30 min and incubated with anti-HP1y (#sc-398562, Santa Cruz Biotechnology) or anti-KI67 (#ab16667, Abcam) primary antibody overnight at room temperature in a humidified chamber. Afterwards, cells were washed and incubated with fluorescent anti-mouse or anti-rabbit secondary antibodies (Alexa Fluor 488, Cy3) at room temperature in a humidified chamber for 1 h. Samples were washed with PBS, and stained with DAPI (#D9542, Sigma-Aldrich) and Phalloidin (#A12379, Thermo Fisher Scientific) for 30 min. Finally, mounting medium (glycerol:PBS 6:4) was applied to the coverslip, sealed with nail polish, and cells were examined using a fluorescence microscope (Olympus BH-2 CCD). Images were taken at three different fluorescence channels: the red one for phalloidin, the blue one for DAPI, and the green one for the antibody of interest. Number of foci and cell area was quantified using ImageJ software.

### Flow cytometry

Caco-2, Caco-2 CXR, SW48, and SW48 CXR (1 × 10^6^) cells were seeded in six-well plates. After overnight starvation, cells were treated for 2 days, washed once with PBS, and fixed by slowly adding cold ethanol dropwise and then stored at −20 °C overnight. The next day, the samples were incubated for 30 min with Propidium Iodide (BIO-RAD # 1351101) + RNase staining solution and analyzed by FACS through CytExpert software.

### Western blot

SW48 and SW48 CXR (5 × 10^4^) cells were seeded in T-25 flasks in 10% FBS medium. After 4 days of treatment, cells were washed and proteins were extracted with RIPA buffer supplemented with a protease inhibitor cocktail (P8340, Sigma-Aldrich, 1:100) and Na_3_VO_4_ (1 mM). Proteins were resolved by sodium dodecyl sulfate (SDS)-polyacrylamide gel electrophoresis, then transferred to a nitrocellulose membrane (Amersham Protran Premium 0.45 μm 300 mm × 4 m). After blocking for 60 min using 3% milk in TBS-T (0.05% Tween-20), the membrane was incubated overnight (4 °C) with primary antibodies. The following primary antibodies were used: anti-HP1y mouse monoclonal antibody (1:500 dilution; sc-398562, Santa Cruz Biotechnology), anti-p-yH2AX mouse monoclonal antibody (1:500; sc-517348, Santa Cruz Biotechnology), anti-SNAI1 mouse monoclonal antibody (1:800; sc-271977, Santa Cruz Biotechnology), anti-GAPDH (14C10) rabbit monoclonal antibody (1:1000, #2118 Cell Signaling Technology). Protein presence was detected through the incubation with anti-rabbit or anti-mouse horseradish-peroxidase-labeled secondary antibody (Dako EnVision + System-HRP Labelled Polymer) followed by chemiluminescent reaction (Clarity Western ECL Substrate, Bio-Rad). Images were acquired and analyzed using ImageLab software.

### 3D spheroid assay

To avoid cellular adhesion and allow anchorage-independent growth, six-well plates were covered with a layer of agar 0.6%. Agar 1.8% was autoclaved and diluted to 0.6% with full medium; 2 ml of 0.6% agar was used to cover each well and left to dry before seeding cells. In each well, 10,000 cells were seeded in 2 ml of 10% FBS medium, supplemented with EGF (10 ng/ml). Treatments were added immediately, according to the information included in the figure legends. After 14 days of treatment, pictures of non-overlapping fields for each well were collected via microscopy at 10× magnification. Spheroids of each picture were counted, and the length of the major and minor axis of each spheroid was measured using ImageJ Software. Axis values below 60 A.U. (Arbitrary Unit) were excluded as not corresponding to mature spheroids, and volume was calculated applying the sphere-adapted formula (major axis × minor axis)^2^/2. Each experiment was repeated three times.

### RNA isolation and qRT-PCR

Caco-2 and Caco-2 CXR cell lines were seeded in six-well plates in 2 ml of 10% FBS medium, whereas SW48 cell lines were seeded in T-25 flasks in 4 ml of 10% FBS medium. Prior to RNA isolation, treatments were added and then removed after 2 days. RNA was extracted using QIAzol Reagent (Invitrogen, Life Technologies), chloroform for separation of three phases, isopropanol for RNA precipitation, and ethanol 75% for washing. RNA was resuspended in DEPC water. Total RNA quantity and quality were determined using a NanoDrop spectrophotometer (Thermo Fischer Scientific, Waltham, MA, USA). RNA reverse transcription (RT) was carried out using the High-Capacity RNA-to-cDNA Kit (Applied Biosystems) and incubating in a thermal cycler (MinicyclerTM PTC-150, MJ Research). Real-time qPCR analysis was performed with Maxima SYBR Green qPCR Master Mix 2× (Fermentas, Thermo Fisher Scientific) in a C1000 Thermal Cycler (Biorad, California, USA) coupled with the CFX96TM Real-Time PCR Detection System (Bio-Rad). *GAPDH* gene was employed as loading control and used to normalize cDNA values. DDCT was calculated, and each gene value was linearized to time zero using the formula 2^−(DDCT)^.

### Statistical analysis

Significance was assessed using *t*-test or one/two-way ANOVA with Sidak’s, Dunnett’s, or Tukey’s multiple comparisons test (****p < 0.0001, ***p < 0.001, **p < 0.01, *p < 0.05).

## Results

### Treatment with a recombinant decoy neutralizing IL-1 (TRAP IL-1) restores drug sensitivity by inhibiting cell growth in CTX-resistant colorectal cancer cell lines

**Fig. 1:**
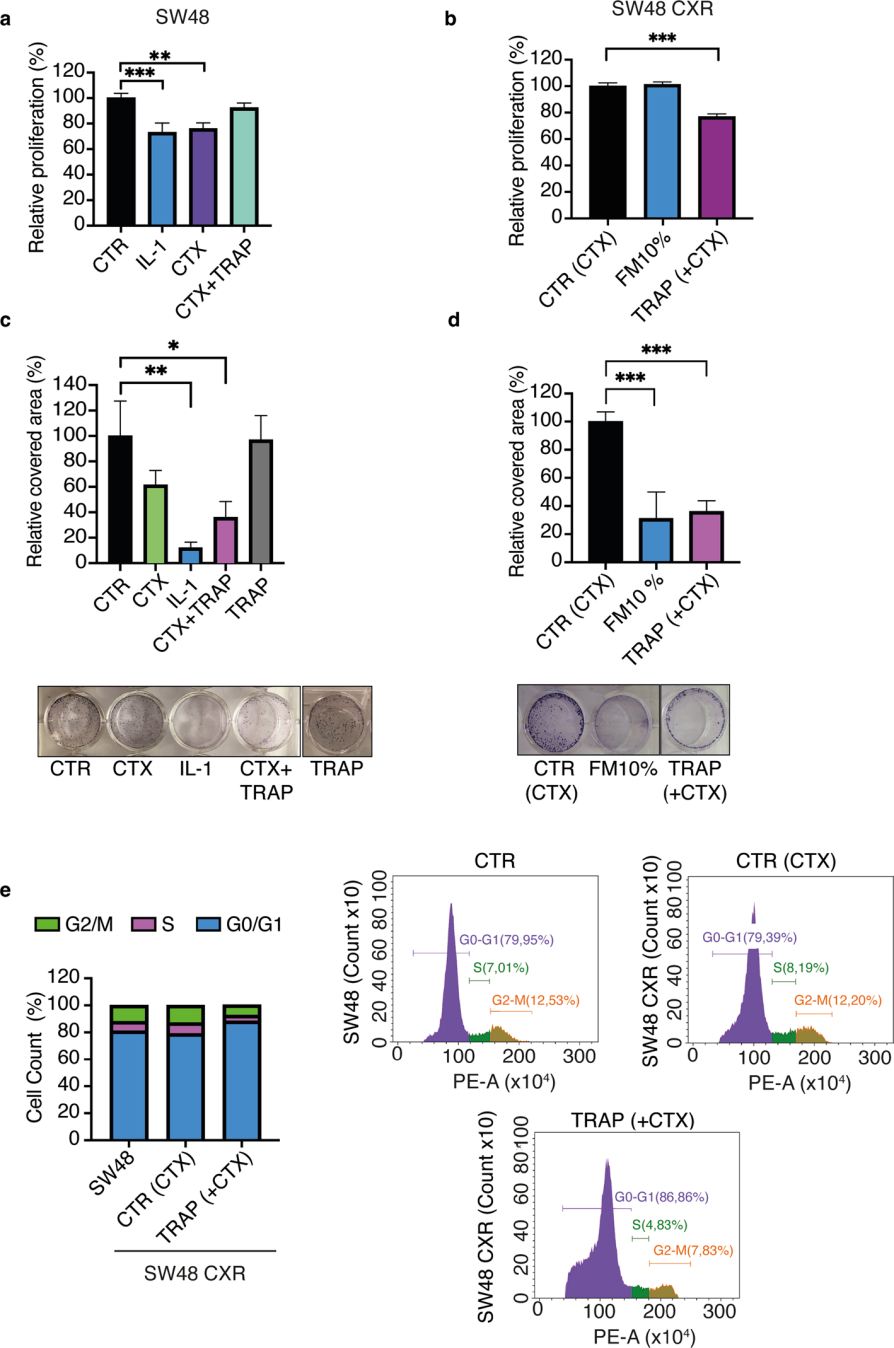
TRAP IL-1 blocks cell cycle, proliferation, and clonogenicity in CTX-resistant SW48. SW48 and SW48 CXR cells (2 × 10^3^) were seeded in 96-well plate. The day after SW48 cells were treated with control medium, IL-1 (α and β, 10 ng/ml each), CTX (50 μg/ml) either alone or in combination with TRAP IL-1 (20 μg/ml). SW48 CXR cells were treated with control cetuximab (50 μg/ml), only medium (10% FBS), or TRAP IL-1 (TRAP 20 μg/ml). After 5 days from seeding, cell viability was assessed through AlamarBlue assay. Histograms show results obtained from SW48 (**a**) and SW48 CXR (**b**). Statistical analysis was carried out using one-way ANOVA and significance determined with respectively Dunnett’s and Tukey’s multiple comparisons test **p < 0.01, ***p < 0.001. To measure clonogenicity, 1 × 10^4^ of SW48 and SW48-CXR were seeded in 12-well plates in triplicate and treated after 24 h as described in **a** and **b**. After 10 days, cells were fixed in 4% PFA and stained with crystal violet for 30 min. The ability of cells to grow in a colony was determined by counting them through ImageJ software. Percentage of covered area is shown for SW48 (**c**) and SW48-CXR (**d**). Statistical analysis was carried out using one-way ANOVA and significance calculated with Dunnett’s multiple comparisons test *p < 0.05, **p < 0.01, ***p < 0.001. (**e**) Cell cycle of SW48 and SW48-CXR (5 × 10^5^) cells treated or not for 48 h with TRAP IL-1 (20 μg/ml) was determined by analyzing DNA content of cells stained with propidium iodide by FACS. Data were processed though Citoflex software

We previously reported that IL-1 α/β cytokine abundance, along with the expression of the receptor IL-1R1, predicts both poor response to CTX and poor relapse-free survival [12, 13]. Here, we employed the SW48 cell line wild-type for KRAS and sensitive to EGFR inhibitors. By exposing these cells to an increasing concentration of CTX for few months, we obtained CTX-resistant SW48 (SW48 CXR) cells. To evaluate the role of the IL-1 family cytokines, we used a recombinant decoy (TRAP IL-1), able to bind both IL-1α and IL-1β isoforms, as previously reported [13]. The use of recombinant decoys has been demonstrated to be an effective strategy to sequester soluble molecules such as growth factors, chemokines, and interleukins. In recent years, several approaches based on this technology have been developed, proving effectiveness by both in vitro and in vivo cancer models [18, 19]. First, we evaluated cell viability under CTX and TRAP IL-1 treatment in SW48 and SW48 CXR cell lines. After seeding in 96-well plate, cells were treated with either medium supplemented with 10% FBS, representing the reference control, IL-1 α and β (10 ng/ml each), CTX (50 μg/ml), or CTX + TRAP IL-1 (TRAP 20 μg/ml) for 4 days. As reported in Fig. 1a, we detected a significant decrease in proliferation when SW48 cells were treated with IL-1 or CTX (Fig. 1a). Importantly, the addition of TRAP IL-1 to CTX blunted its inhibitory action (Fig. 1a), suggesting that cetuximab reduces cell proliferation by inducing IL-1. Conversely, SW48 CXR displayed a different profile. SW48 CXR cells were grown in culture with medium containing 50 μg/ml of CTX, and 24 h after seeding, cells were treated with either CTX (the reference sample), 10% of FBS-containing medium, or a combination of CTX and TRAP IL-1. Under CTX treatment, cells were growing undisturbed, confirming resistance to the CTX antibody; also, no changes were observed when growing in CTX-depleted medium for 4 days (Fig. 1b). However, strikingly, cells treated with the combination of CTX and TRAP IL-1 showed reduced proliferation, thus regaining sensitivity to the antibody (Fig. 1b). A second KRAS WT colon cancer cell line, Caco-2 CTX-resistant (Caco-2 CXR), displayed a similar trend when TRAP IL-1 was added to the medium in combination with CTX (Additional file 1: Fig. S1a). Next, we performed a colony formation assay in sensitive and resistant SW48 cells, to evaluate the capability of TRAP IL-1 to restore CTX sensitivity, in a long-time experimental setting. In 10 days of treatment, IL-1 as well as CTX strongly reduced the number of colonies formed by SW48 cells (Fig. 1c). Indeed, significant reduction of cell growth was observed in the cells co-treated with TRAP IL-1 suggesting that IL-1 secreted under CTX treatment strongly influences drug sensitivity, already after 10 days exposure (Fig. 1c). Strikingly, IL-1 involvement in CTX-response was also confirmed in SW48 CXR and Caco-2 CXR cells. Indeed, by removing CTX from the medium, we observed a pronounced reduction of colony formation, confirming the dependency of resistant cells to the drug, and once again, the combination with TRAP IL-1 strongly impaired proliferation and the effect was significant in both resistant cell lines (Fig. 1d and Additional file 1: Fig. S1b).

Thereafter, to explore the mechanism of action, we evaluated cell cycle distribution in Caco-2 following 48 h of treatment with IL-1, CTX, and CTX + TRAP (Additional file 1: Fig. S1c). Under IL-1 treatment, and to a lesser extent CTX, Caco-2 cells showed an increase in G0/G1 phase and a simultaneous reduction of the S and G2/M phases (Additional file 1: Fig. S1c). Importantly, addition of TRAP IL-1 to CTX rescued the cell cycle distribution (Additional file 1: Fig. S1c). A completely different scenario was observed in CTX-resistant cell lines of both SW48 CXR and Caco-2 CXR (Fig. 1e and Additional file 1: Fig. S1d). Clearly, the addition of TRAP IL-1 induced cell cycle arrest in G0/G1 phase compared with the control sample, represented by cells under CTX treatment (Fig. 1e and Additional file 1: Fig. S1d), confirming the results of the proliferation assays, (Fig. 1d). In conclusion, the “CTX-addiction” of resistant cells seems to rely on IL-1 pathway activation, and IL-1 sequestering is essential for impairing cell cycle activity and proliferation.

### TRAP IL-1 abolishes the increase in cell size induced by CTX in responsive cells

**Fig. 2:**
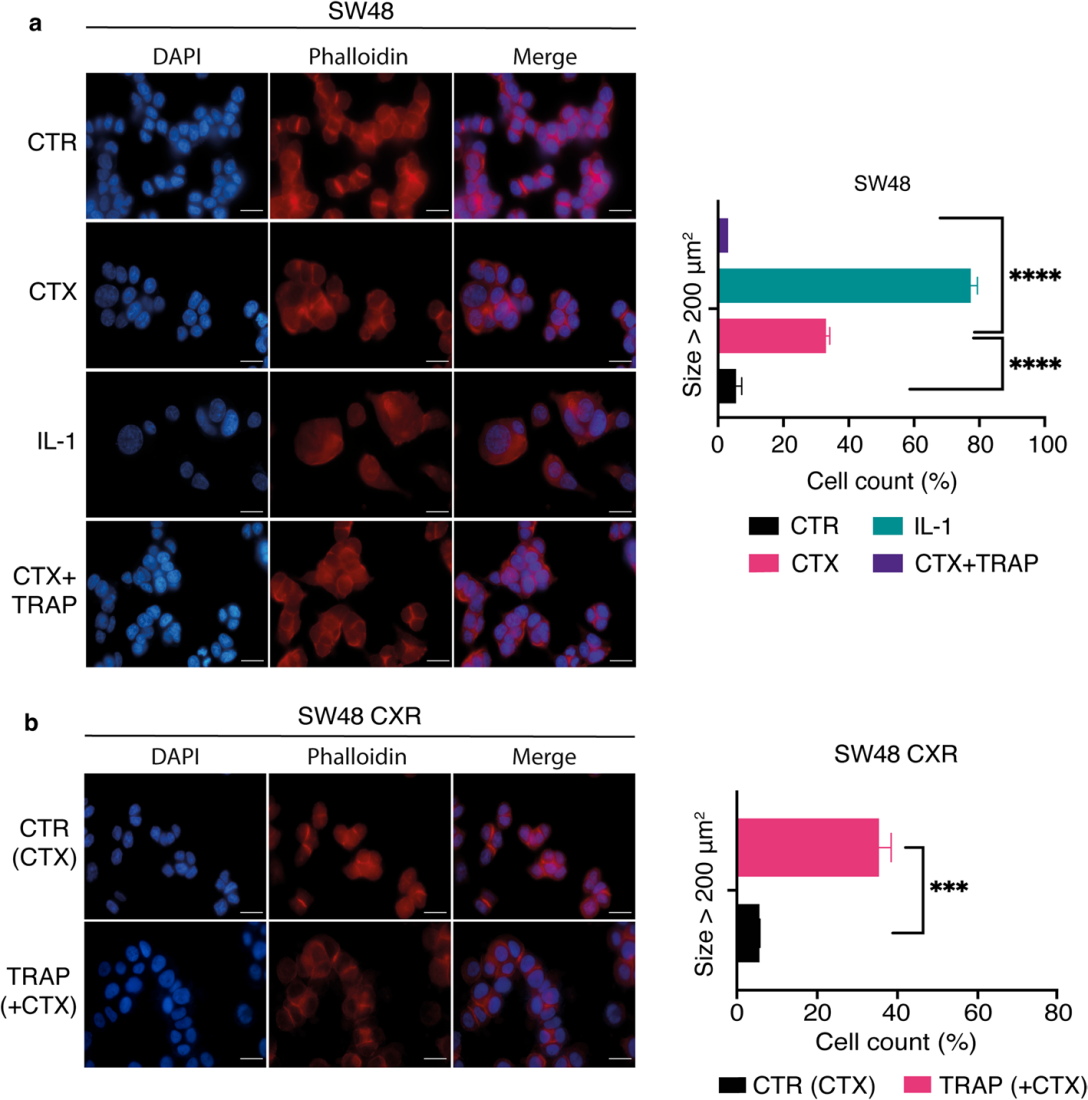
TRAP IL-1 inhibits CTX-induced gigantism while stimulating senescence morphology in SW48-CXR. SW48 and SW48 CXR cell lines (4 × 10^4^ cells per well) were seeded on 13 mm coverslip. After 24 h, SW48 cells were treated for 4 days with control medium (10% FBS), IL-1 (α and β, 10 ng/ml each), and CTX (50 μg/ml) alone and in combination with TRAP IL-1 (20 μg/ml). SW48 CXR cells were treated with control medium (10% FBS + CTX 50 μg/ml), and TRAP IL-1 (20 μg/ml). Subsequently, they were fixed and stained with DAPI (nuclei) and phalloidin (TRITCH) for 30 min. Finally, coverslips were mounted on slides and examined with a fluorescence microscope. ImageJ software was used to measure cell area of four different images taken for each sample. Percentage of cell number was calculated based on cell size and grouped in cells with area < or > 200 μm^2^, represented by histograms for SW48 (**a**) and SW48 CXR (**b**). Statistical analysis was carried out using two-way ANOVA with Tukey’s and Sidak’s multiple comparisons test, respectively, ***p < 0.001, ****p < 0.001. Scale bar, 20 mm

Senescence is a mechanism of stress response and can be activated by several damaging stimuli, which ultimately lead to cell cycle arrest. One of them is represented by the production of cytokines (such as IL-1, IL-6, or IL-8) or chemokines, known as SASP [20, 21]. However, senescent cells have heterogeneous phenotype and different markers can be used to detect them [14, 22–24]. Along with cell cycle arrest, another important feature is their irregular shape and an enlarged cell body [25]. Thus, to analyze cell size in vitro, we employed immunofluorescence, in SW48 cells, targeting actin microfilaments and nuclei through TRITC-conjugated phalloidin and DAPI staining, respectively (Fig. 2a). We observed that the control cells presented a wide homogeneity in cell sizes, with almost 100% of cells having an area less than 200 μm^2^. On the other hand, cells treated with IL-1 α/β (10 ng/ml each) or CTX (50 μg/ml) showed an increased heterogeneity, with a distinct cell subpopulation exhibiting an enlarged and flat morphology and having an area > 200  μm^2^, with abundant cytoplasm and irregular nuclei, reported with the relative quantification in Fig. 2a. Interestingly, this phenotype appeared highly plastic since the co-treatment with TRAP IL-1 (20 μg/ml) was able to revert the effect of CTX on cell size (Fig. 2a). The same evaluation was performed for SW48 CXR. Cells growing with CTX (reference sample) displayed a homogeneous population, and about 100% of the CTX-resistant cells displayed a size below 200 μm^2^. Conversely, TRAP IL-1 treatment drastically increased cell size, with about 40% of the cells exhibiting an expanded phenotype (Fig. 2b). To sum up, sequestering IL-1 in the medium of resistant cells strongly increases the cellular size, by reverting to a senescence-like phenotype.

### Cetuximab decreases cell proliferation, induces EGFR degradation, and increases HP1y protein expression during short-time treatment

**Fig. 3:**
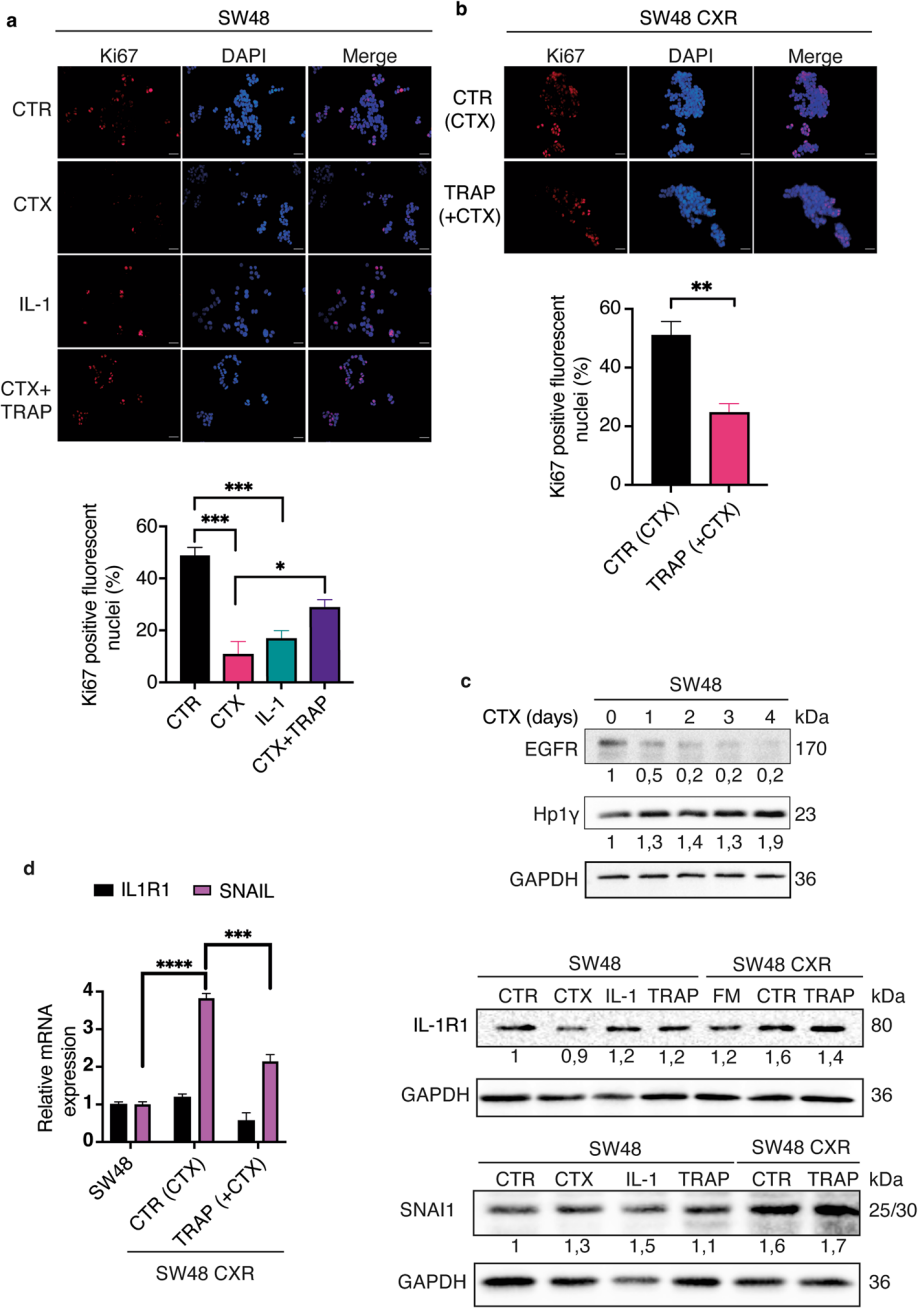
SW48-CXR exhibits a lower number of Ki67-positive cells when treated with TRAP IL-1 and higher IL-1R1 and SNAI1 protein expression compared with SW48. Sensitive and resistant SW48 cell lines were treated for 4 days as follows: SW48 with control medium (10% FBS), IL-1 (α and β, 10 ng/ml each), and CTX (50 μg/ml) alone and in combination with TRAP IL-1 (20 μg/ml) (**a**), while SW48 CXR cells were treated with control medium (10% FBS + CTX 50 μg/ml) and TRAP IL-1 (20 μg/ml) (**b**). Cells were incubated with anti-Ki67 antibody and with DAPI (nuclei). Number of Ki67-positive nuclei was calculated through ImageJ software from three different fields taken for each sample. Histograms show the percentage of Ki67-positive nuclei of one representative of two independent experiments. Statistical analysis was carried out using two-way ANOVA with Tukey’s multiple comparisons test and *t*-test in **a** and **b**, respectively, *p < 0.05, **p < 0.01, ***p < 0.001. Scale bar, 29 mm. In the upper panel, SW48 cells were seeded in flask and treated with CTX (50 μg/mL) from day 0 to day 4. Immunoblots were processed with the indicated antibodies. In the lower panel, SW48 cells were treated with control medium, IL-1 (α and β 10 ng/ml each), CTX (50 μg/ml), and TRAP IL-1 (20 μg/ml), and SW48 -CXR cells were treated with control medium (FM, with CTX 50 μg/ml) and TRAP IL-1, for 4 days. Protein extracts were incubated with anti IL-1R1 and SNAI1. Signals were quantified and normalized (numbers below each lane). GAPDH was used as loading control protein (**c**). SW48-CXR cells were maintained under CTX treatment (as described in “Materials and methods”). The next day, medium was changed, and the indicated treatment was applied (TRAP IL-1 20 μg/ml) for 2 days. Thereafter, mRNA was extracted, and gene expression was determined for* IL1R1* and *SNAI1* (**d**). Statistical analysis was carried out using two-way ANOVA with Sidak’s multiple comparisons test ***p < 0.001; ****p < 0.001

CTX is a well-known chimeric monoclonal antibody widely used in the clinic for patients with advanced colorectal cancer. It binds with high affinity to the extracellular domain III of EGFR blocking downstream signalling pathways, inducing antibody-dependent cellular cytotoxicity (ADCC), and promoting receptor internalization and degradation [26]. CTX activity on the cell cycle was evaluated by measuring the expression of Ki67 protein, through immunofluorescence. Results obtained in the SW48 sensitive cell line confirmed that, under CTX and IL-1 treatment, cells showed fewer Ki67-positive nuclei compared with control sample (Fig. 3a). This effect was only partially restored by TRAP IL-1, which, conversely, induced a significant reduction of Ki67 expression in SW48-CXR (Fig. 3b). Subsequently, through western blot, we confirmed the ability of CTX to reduce EGFR protein levels in the SW48 cell line, treated at different timepoints from day 0 to day 4 (Fig. 3c). CTX maintained a low level of EGFR expression over time, likely driving the receptor to degradation as previously reported [27]. Next, we evaluated the expression of the senescence marker HP1y, a structural protein present in the chromatin foci. Immunoblotting revealed a higher level of HP1y already after 24 h of CTX treatment compared with the control cells (time 0) (Fig. 3c). Finally, in sensitive and resistant SW48 cell lines, we determined the levels of IL-1 receptor (IL-1R1) and the expression of SNAI1, a transcriptional repressor controlling the EMT transition by repressing E-cadherin [28] (Fig. 3c). Consistent with the increase in IL-1 cytokine abundance that we previously reported [12], here we detected higher levels of the IL-1R1 at basal growth conditions, in CTX-resistant cells compared with parental cells (Fig. 3c). Moreover, in SW48 CXR cells, the level of IL-1R1 decreased when cultured without CTX (Fig. 3c), and SNAI1 showed an increased expression in resistant cells compared with parental ones (see Additional Figure 3, Figure S3 for the original blots). Finally, using real-time analysis, we tested the expression of *SNAI1* and *IL-1R1* on SW48 and SW48 CXR (Fig. 3d). Strikingly, CTX-resistant cells displayed higher expression level of *SNAI1* compared with sensitive cells. Interestingly, when TRAP IL-1 was added, both *SNAI1* and *IL-1R1* mRNA expression levels decreased. These results suggest that, in sensitive cells, CTX exerts its action through the activation of a senescence program, as revealed by both cell size and HP1y abundance evaluation. However, resistant cells are reprogrammed towards an EMT/stemness phenotype. To further investigate these properties, we cultured Caco-2 cells in low-attachment conditions. This test specifically provides information about cells’ aptitude to proliferate in total absence of adhesion support, a property correlated with cell stemness. Indeed, Caco-2 cells growing in suspension may organize themselves into spherical 3D structures, namely spheroids or colonospheres. We evaluated both sensitive and resistant cells and tested the number and the size of spheroids under CTX treatment, alone or in combination with TRAP IL-1. In line with the model, CTX and IL-1 treatments were able to impair spheroids growth, and addition of TRAP IL-1 was not sufficient to reverse this effect. On the other hand, Caco-2 CXR spheroids appeared much bigger compared with the sensitive conditions and TRAP IL-1 significantly reduced spheroids’ size. These data are provided in Additional file 2: Fig. S2.

### TRAP IL-1 inhibits HP1y nuclear foci formation by CTX treatment in SW48 cell line and restores senescence in CTX-resistant cells

**Fig. 4:**
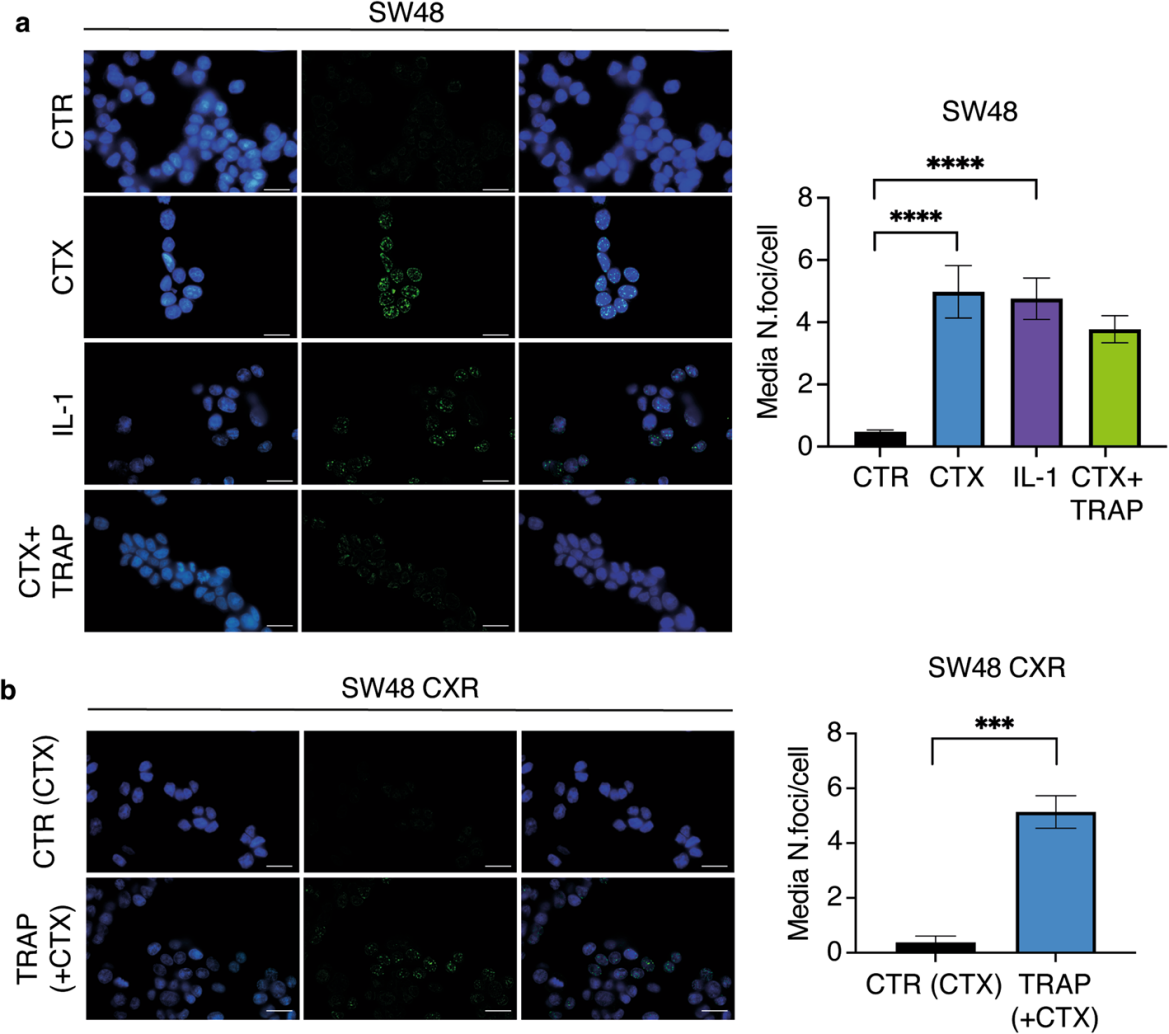
TRAP IL-1 decreases HP1y foci number in SW48, while increasing it in SW48 CXR. Sensitive and resistant SW48 cell lines (4 × 10^4^ cells per well) were seeded on 13 mm coverslip. Twenty-four hours later, SW48 cells were treated for 4 days with control medium (10% FBS), IL-1 (α and β, 10 ng/ml each), and CTX (50 μg/ml) alone and in combination with TRAP IL-1 (20 μg/ml) (**a**), while SW48 CXR cells were treated with control medium (10% FBS + CTX 50 μg/ml), and TRAP IL-1 (20 μg/ml) (**b**). After fixation, permeabilization, and blocking with 2% BSA, cells were incubated with anti-HP1y antibody. The next day, cells were washed, incubated with secondary antibody FITC mouse, and stained with DAPI (nuclei). Finally, coverslips were mounted on slides and examined under a fluorescence microscope. Number of HP1y foci was determined and analyzed through ImageJ software from three different fields for each sample. Histograms shows the average of HP1y foci for each cell. Statistical analysis was carried out using two-way ANOVA with Tukey’s multiple comparisons test ***p < 0.001, ****p < 0.0001. Scale bar, 20 mm

**Fig. 5:**
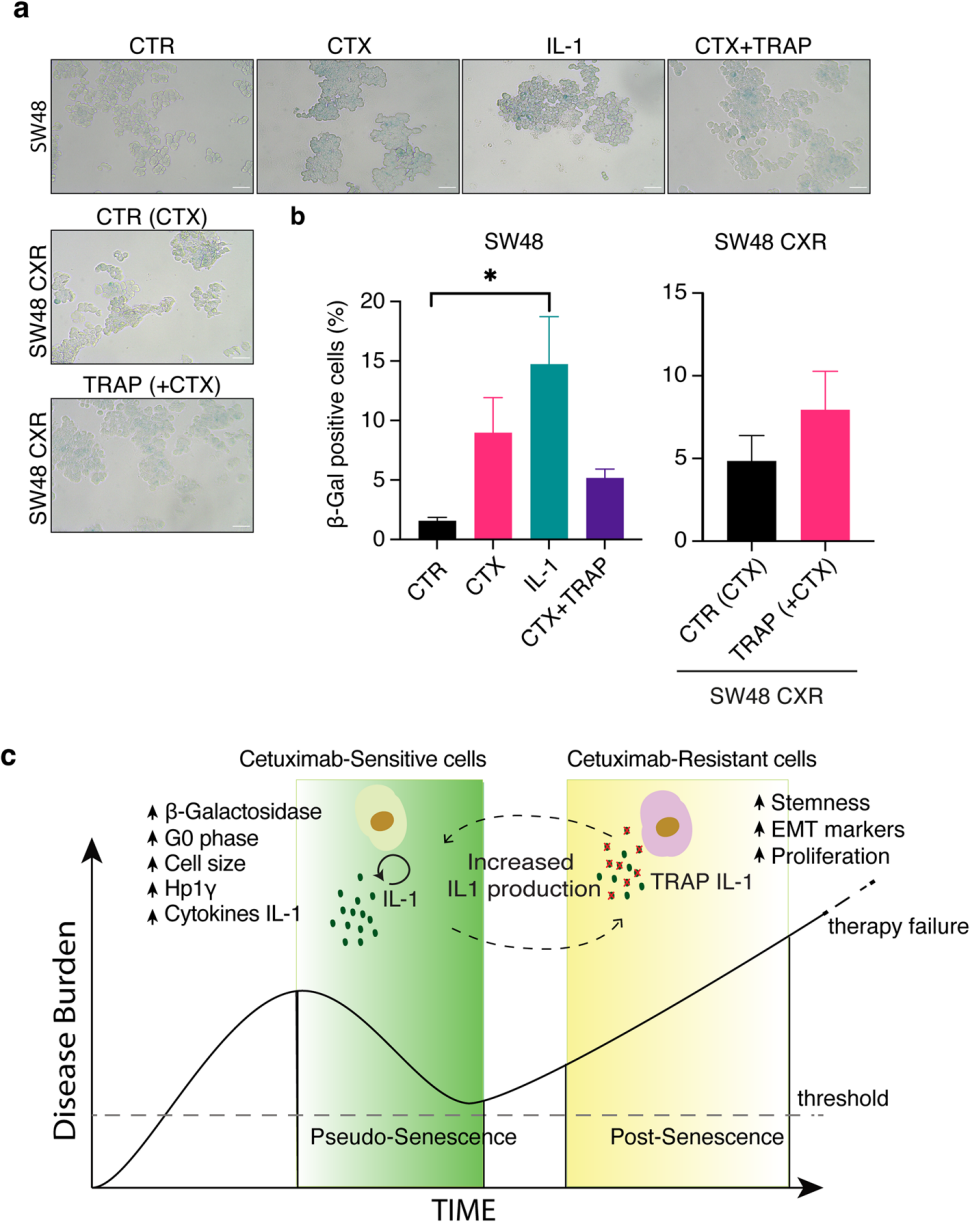
TRAP IL-1 blocks CTX-induced β-galactosidase activity in SW48 while increasing it in SW48 CXR. SW48 and SW48 CXR cells were seeded in 12-well plates in 10% of FBS medium and treated the following day with CTX (50 μg/ml), IL-1 (α + β 10 ng/ml each), and TRAP (20 μg/ml). After 4 days, cells were washed, fixed, and stained with β-galactosidase for 5 h at 37 °C. Photos were taken at 20× magnification (Leica). Scale bar, 50 μm (**a**). The histograms show analysis of positively stained cells (mean ± SEM) for SW48 and SW48 CXR, respectively (**b**). Statistical analysis was carried out using one-way ANOVA with Tukey’s multiple comparison test. (**c)** The model depicted in the cartoon describes how, in the CTX-sensitive phase, IL-1 induces pseudo-senescence and growth-arrest, but in resistant cells, IL-1 pushes cells into a post-senescence stage

Senescence-associated heterochromatin foci (SAHF) are believed to be involved in cell cycle exit of senescent cells by inhibiting the expression of specific *cyclin* genes. One of the heterochromatin-forming proteins present in the nuclear foci SAHF is heterochromatin protein 1 (HP1). In cells entering senescence, HP1y isoform is phosphorylated for efficient incorporation into SAHF [29]. Once we confirmed the presence of HP1y protein by immunoblot (Fig. 1c), we determined if the protein localized in the DNA foci. Thus, we proceeded by staining SW48 and SW48 CXR for HP1y (green) and nuclei (DAPI), after 4 days of treatment (Fig. 4a). Images taken by a fluorescence microscope showed that, in SW48 cells, both samples treated with IL-1 α/β (10 ng/ml each) or CTX (50 μg/ml) displayed higher number of HP1y foci in the nucleus compared with the control, whereas the co-administration of TRAP IL-1 inhibited CTX-induced nuclear foci formation (Fig. 4a). The same experiment was performed with SW48 CXR, which probed negative for HP1y foci under CTX treatment. In these cells, sequestering IL-1 from the medium increased the HP1y foci staining (Fig. 4b). Thus, in resistant cells, TRAP IL-1 treatment induced chromatin rearrangement, forming dense structure characteristic of senescent cells. Our results indicated that, in sensitive cells, treatment with CTX is correlated with an increased IL-1 production, greater cell size, and higher HP1y protein expression and nuclear foci localization. Considering that IL-1 is also one of the main molecules of SASP implicated in the onset of senescence, we confirmed the correlation between CTX and senescence, by evaluating the β-galactosidase activity. For this experiment, SW48 and SW48 CXR cells were treated with CTX (50 μg/ml), IL-1α + β (10 ng/ml), or TRAP IL-1 (20 μg/ml). After 4 days, cells were fixed and stained for β-galactosidase marker. Analysis of staining revealed, in SW48 cells, an increase in β-galactosidase activity under both CTX treatment and IL-1 stimulation (Fig. 5a and b). On the other hand, TRAP IL-1 treatment was able to revert resistant cells to a senescence phenotype with consequent growth arrest (Fig. 5a and b).

## Discussion

Epidermal growth factor receptor (EGFR)/BRAF inhibition by CTX represses several DNA repair genes with consequent induction of DNA damage [30] and cellular senescence [31]. In this regard, we previously reported a correlation between resistance to CTX and increased IL-1 expression [12, 32]. A mechanism of therapy-induced secretomes mediating resistance has been previously reported in melanoma and lung cancer [33].

For example, chemotherapy-induced senescence in ovarian cancer cells appeared responsible for a cancer-promoting phenotype [34]. Here, we proved that IL-1 secretion induced by CTX is driving a senescence-like phenotype, which leads to cell cycle arrest. Intriguingly, CTX activity seems to be dependent on the production of the senescence inducer IL-1. Indeed, immediately after CTX exposure, CRC cells activate a cell arrest program dependent on IL-1 secretion. Secondarily, upon acquisition of resistance, IL-1 availability is associated with cell regrowth. Paradoxically, IL-1 seems to exert two opposite roles: in sensitive cells, it promotes growth arrest by senescence, while in resistant cells it promotes cell growth.

This model was validated by using a recombinant decoy that is effective in sequestering IL-1 in the medium of two different colon cancer cell lines, sensitive to EGFR inhibition by CTX. Indeed, upon CTX treatment, CRC cells showed inhibition of proliferation or clonogenicity and underwent senescence, consequent to IL-1 secretion (Fig. 5c). In line with this finding, TRAP IL-1 blunted CTX activity, thus clearly proving that CTX-induced cell cycle arrest is mediated by IL-1 production. Senescence appears to be the mechanism by which IL-1 influences the cellular response to CTX in sensitive cells (Fig. 5c). Senescent cells chronically exposed to a pro-inflammatory environment may promote a process known as post-senescence, characterized by cell adaptation and stemness with tumor progression [35–37]. Interestingly, in our hands, CTX-resistant cells confirmed the acquisition of stemness properties and the rescue of DNA damage, a process known to be dependent on p53 and its partner p63, which might also lead to stemness [38].

IL-1 neutralization by TRAP IL-1 was sufficient to restore the senescence-mediated growth arrest. These results indicate the relevance of IL-1 and the downstream pathway in CTX resistance and suggest a mechanism driven by IL-1 in the establishment of CTX resistance. The cytokine secreted by both cancer and noncancer cells creates a pro-inflammatory microenvironment, which brings cells into “pseudo-senescence.” In such a state, under chronic IL-1 exposure, cells are able to escape drug inhibition, acquire stem-like properties, and reactivate into a fully adapted and drug-addicted phenotype that ultimately drives cancer recurrence. If correct, we predict that, in vivo, cetuximab stimulates the recruitment of immune infiltrates secreting IL-1, capable of eradicating cancer cells through a bystander effect. Secondarily, a fraction of a cancer cells escapes the senescence growth arrest/death, gaining the capability to proliferate. Intriguingly, at this stage, cancer cells appear addicted to the IL-1 factors, enabling cancer progression under monoclonal antibodies. Interestingly, IL-1 inhibition remains lethal for those cells, representing an acquired actionable target.

## Conclusions

Overall, employing senolytic agents may represent a powerful tool to fighting post-senescent resistant cell progression. This has already been suggested for premalignant lesions [39] and is corroborated by the clinical data showing a drastic reduction in lung cancer incidence among patients with prior myocardial infarction treated with the antibody neutralizing IL-1β, canakinumab [40]. Our results suggest that pharmacologically targeting IL-1 may overcome resistance to monoclonal antibodies and disease recurrence. For this reason, we endorse the “one–two punch model,” suggested by Bernards’ laboratory [41]. Indeed, anti-EGFR neutralizing antibodies may function as first punch to slowing down cancer progression by inducing senescence, and next, the employment of a senolytic agent targeting IL-1 will restore the sensitivity to cetuximab in patients showing recurrence during therapy. To sum up, an adjuvant neutralization of IL-1 under CTX treatment may constitute a future strategy specifically for patients who proved refractory to CTX monotherapy.

## Supplementary Information

**Additional file 1: Figure S1.** TRAP IL-1 blocks proliferation and clonogenicity in CTX-resistant Caco-2 cell line. Caco-2 CXR cells (1 × 10^3^) were seeded in 96-well plate. The next day, Caco-2 CXR cells were treated with control medium supplemented with CTX (10 μg/ml), only medium (FM 10% FBS), and TRAP IL-1 (20 μg/ml). After 5 days, cell viability was assessed through AlamarBlue assay. Histograms show the average of two independent experiments quintuplicated (**a**). To measure clonogenicity, 3 × 10^3^ of Caco-2 CXR were seeded in 12-well plates in triplicate and treated after 24 h as described in **a** and **b**. After 10 days, cells were fixed in 4% PFA and stained with crystal violet for 30 min. The ability of cells to grow in a colony was determined by analyzing the covered area through ImageJ software. Percentage of covered area is shown (**b**). Statistical analysis was carried out using one-way ANOVA and significance calculated with Tukey’s multiple comparisons test *p < 0.05, **p < 0.01. Cell cycle of Caco-2P (c) and Caco-2 CXR (d) cells was determined with the indicated treatment analyzing DNA content of cells, stained with propidium iodide by FACS. Data were processed through Citoflex software

**Additional file 2: Figure S2.** TRAP IL-1 decreases spheroids’ size in Caco-2 CXR cells. Soft agar colony formation assay was performed in Caco-2P (A) and Caco-2 CXR (B). Cells (1 × 10^4^) were overlaid on 0.6% agar in six-well plates and suspended in medium containing the following treatments: control medium, CTX (10 μg/ml) and CTX + TRAP IL-1 (20 μg/ml) for CACO-2P; control medium (CTX, 10 μg/ml) and TRAP IL-1 for Caco-2 CXR. After 2 weeks, spheroids were analyzed and photographed at 10× magnification. Scale bar, 70 μm. Scatter plots show spheroid size calculated from three different images taken for each well. Statistical analysis was carried out using one-way ANOVA with Tukey’s multiple comparison test. ***p < 0.001, ****p < 0.0001

**Additional file 3: Figure S3.** Original blots are provided.

## Data Availability

All data generated or analyzed during this study are included in this published article (and its additional information files).

## Abbreviations

CRC: Colorectal cancer
mAbs: Monoclonal antibodies
CTX: Cetuximab
IL-1: Interleukin-1
IL-1R1: IL-1 receptor 1
SASP: Senescence associated-secretory phenotype
TRAP IL-1: Recombinant decoy neutralizing IL1α/β
SW48 CXR: Resistant SW48
Caco-2 CXR: Resistant Caco-2
SAHF: Senescence-associated heterochromatin foci
