# Supplementary figures and images for: Senescence-associated reprogramming induced by interleukin-1 impairs response to EGFR neutralization
Source: Cell Mol Biol Lett. 2022 Mar 2;27:20. doi: 10.1186/s11658-022-00319-7 (PMC8903543; doi:10.1186/s11658-022-00319-7)

**a**

Caco-2 CXR

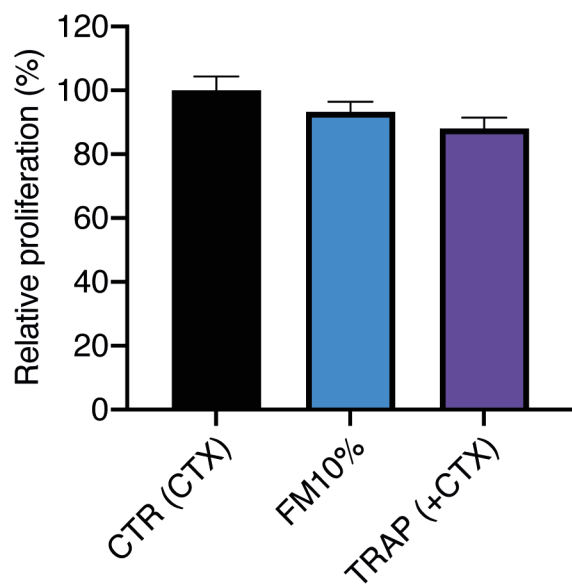**b**

Caco-2 CXR

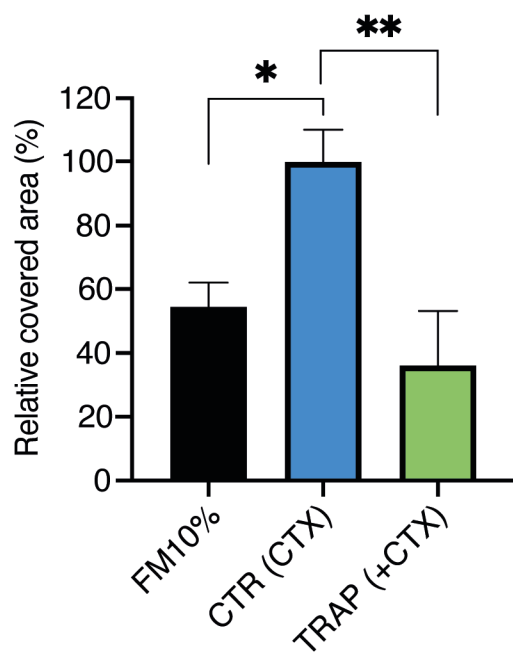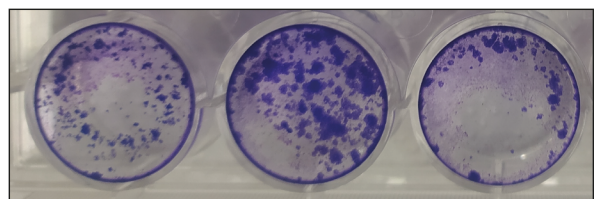

FM10%

CTR  
(CTX)TRAP  
(+CTX)**c**

Caco-2 P

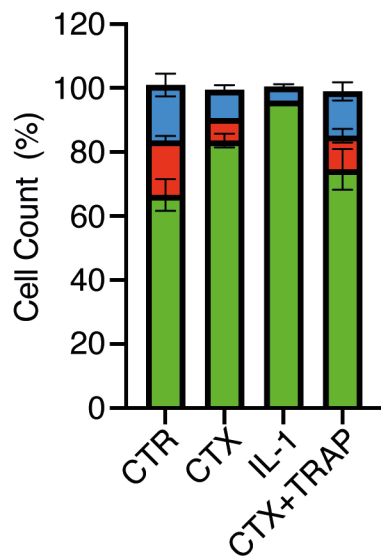**d**

Caco-2 CXR

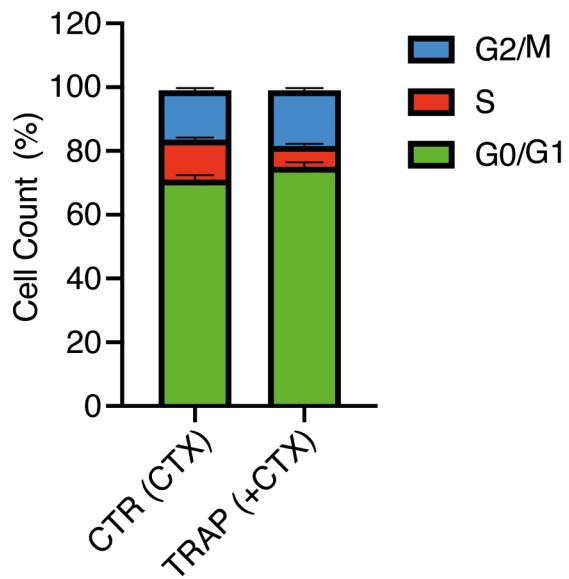

Supplement: Supplementary file 1 — Additional file 1: Figure S1. TRAP IL-1 blocks proliferation and clonogenicity in CTX-resistant Caco-2 cell line. Caco-2 CXR cells (1 × 10^3^) were seeded in 96-well plate. The next day, Caco-2 CXR cells were treated with control medium supplemented with CTX (10 μg/ml), only medium (FM 10% FBS), and TRAP IL-1 (20 μg/ml). After 5 days, cell viability was assessed through AlamarBlue assay. Histograms show the average of two independent experiments quintuplicated (a). To measure clonogenicity, 3 × 10^3^ of Caco-2 CXR were seeded in 12-well plates in triplicate and treated after 24 h as described in a and b. After 10 days, cells were fixed in 4% PFA and stained with crystal violet for 30 min. The ability of cells to grow in a colony was determined by analyzing the covered area through ImageJ software. Percentage of covered area is shown (b). Statistical analysis was carried out using one-way ANOVA and significance calculated with Tukey’s multiple comparisons test *p < 0.05, **p < 0.01. Cell cycle of Caco-2P (c) and Caco-2 CXR (d) cells was determined with the indicated treatment analyzing DNA content of cells, stained with propidium iodide by FACS. Data were processed through Citoflex software [file 11658_2022_319_MOESM1_ESM.pdf]

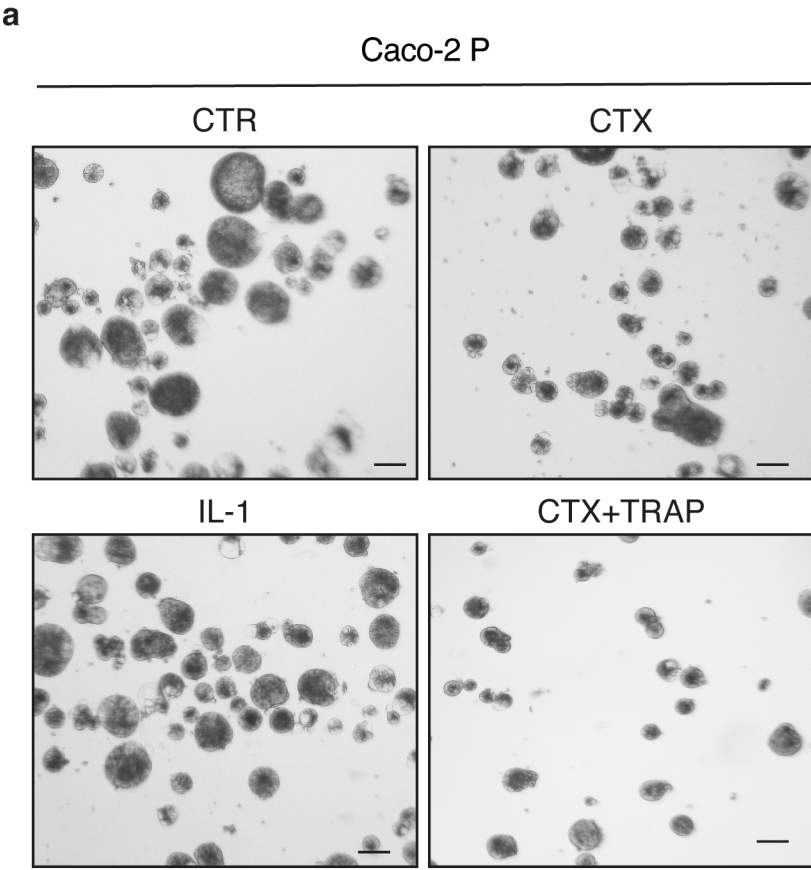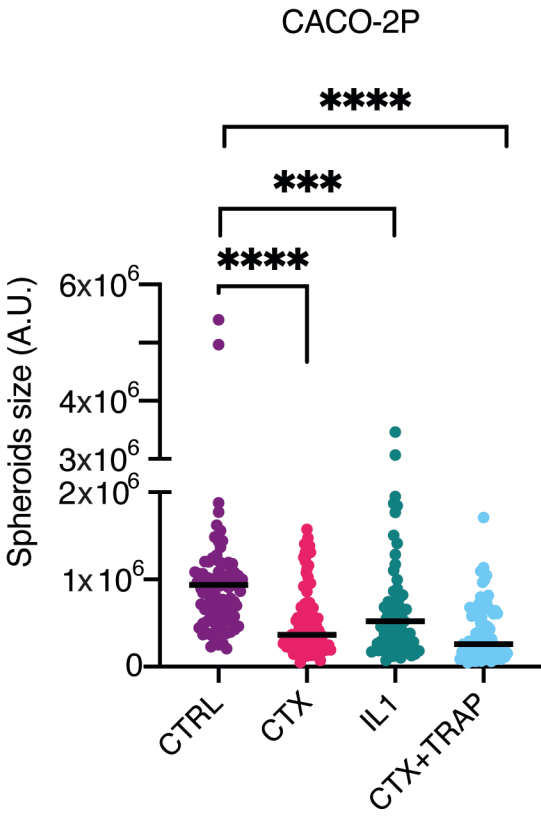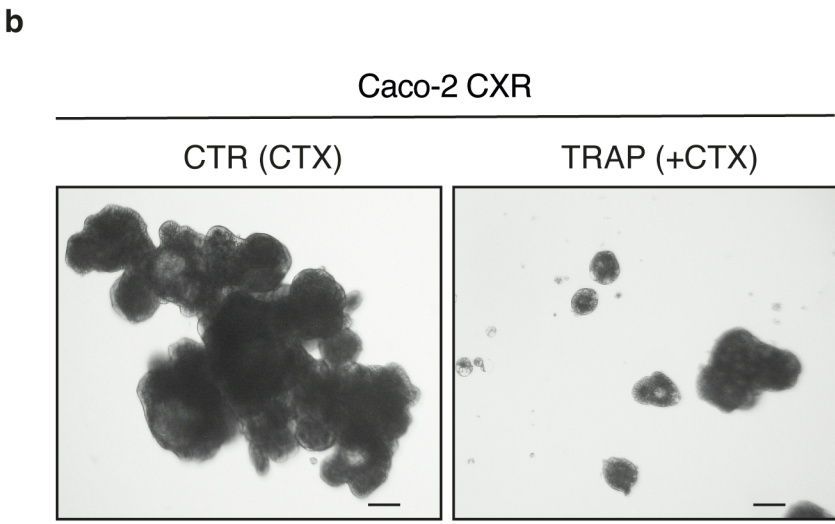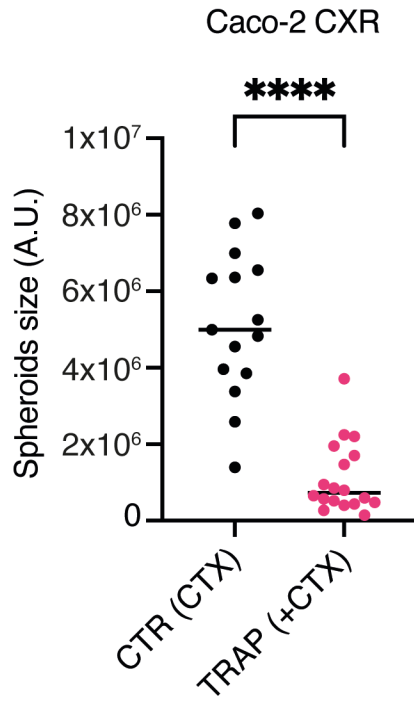

Supplement: Supplementary file 2 — Additional file 2: Figure S2. TRAP IL-1 decreases spheroids’ size in Caco-2 CXR cells. Soft agar colony formation assay was performed in Caco-2P (A) and Caco-2 CXR (B). Cells (1 × 10^4^) were overlaid on 0.6% agar in six-well plates and suspended in medium containing the following treatments: control medium, CTX (10 μg/ml) and CTX + TRAP IL-1 (20 μg/ml) for CACO-2P; control medium (CTX, 10 μg/ml) and TRAP IL-1 for Caco-2 CXR. After 2 weeks, spheroids were analyzed and photographed at 10× magnification. Scale bar, 70 μm. Scatter plots show spheroid size calculated from three different images taken for each well. Statistical analysis was carried out using one-way ANOVA with Tukey’s multiple comparison test. ***p < 0.001, ****p < 0.0001 [file 11658_2022_319_MOESM2_ESM.pdf]

FIG.3A

EGFR

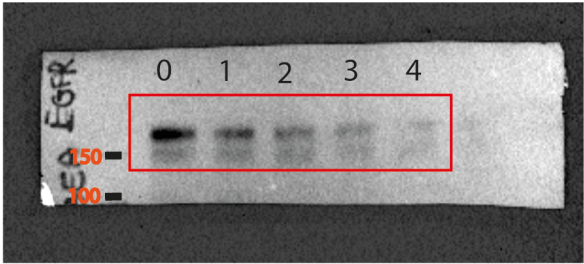

FIG.3B

IL1R1

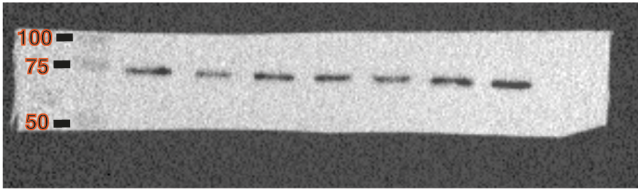

HP1 $\gamma$

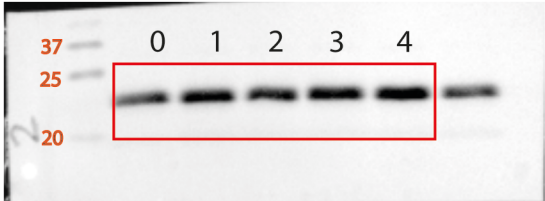

GAPDH

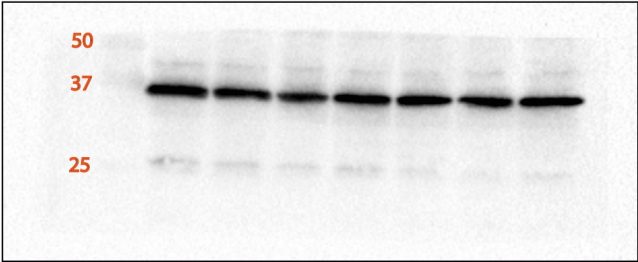

GAPDH

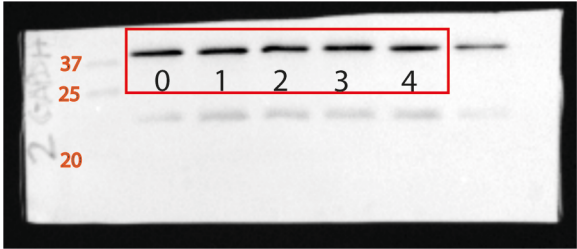

SNAI1

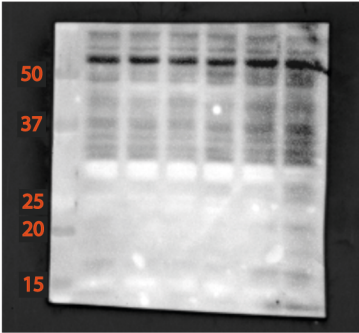

GAPDH

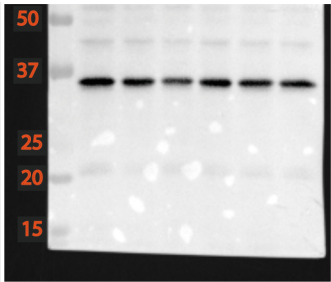

Supplement: Supplementary file 3 — Additional file 3: Figure S3. Original blots are provided. [file 11658_2022_319_MOESM3_ESM.pdf]
